# Knowledge and Attitudes of Blood Donors Toward Sickle Cell Anemia in Ibadan

**DOI:** 10.7759/cureus.70199

**Published:** 2024-09-25

**Authors:** Olanrewaju A Amusat, Abdulhammed O Babatunde, Dimeji A Olawuyi, Deborah A Ogundijo, Zainab O Aderohunmu, Abdul-Gafar O Afolayan, Uthman H Alao, Abdulqudus A Akintola, Abdulrahman O Oseni, Habeeb A Abdulrasheed

**Affiliations:** 1 Urology, Luton and Dunstable University Hospital, Luton, GBR; 2 Urology, University College Hospital, Ibadan, NGA; 3 Medicine and Surgery, Faculty of Clinical Sciences, College of Medicine, University of Ibadan, Ibadan, NGA; 4 Medicine and Surgery, Zenith Medical and Kidney Centre, Abuja, NGA; 5 Biomedical Laboratory Science, Faculty of Basic Medical Sciences, College of Medicine, University of Ibadan, Ibadan, NGA; 6 Medicine and Surgery, UNIOSUN Teaching Hospital, Osogbo, NGA; 7 Urology, University Hospitals Birmingham, Birmingham, GBR

**Keywords:** blood, donor, hbss, sca, sickle cell anemia

## Abstract

Background

Sickle cell anemia (SCA) is a common condition of public health concern in Nigeria. Different therapeutic approaches have been developed to manage SCA, including blood transfusion. In a bid to develop a sustainable solution to the blood supply pool, the SmileBuilders Initiative organizes a blood donation drive (Donate-A-Pint Project) quarterly in Ibadan, Nigeria. This study aimed to assess the awareness, knowledge, and attitudes of blood donors in Ibadan, Nigeria, towards SCA.

Methods

A descriptive cross-sectional study was conducted at the University College Hospital Blood Bank in Ibadan over a period of three months. A validated questionnaire, based on similar studies in Nigeria, was used to collect data from blood donors. The SPSS version 23 was used for data analysis, and the chi-square test of independence assessed associations between variables, with a significance level of 0.05.

Results

Among 205 donors, 68.8% were male, and 42.4% were in the age category of ≥25 years. Most donors (60.0%) were university students. While 12.3% had never heard of SCA, 79.5% were aware of their genotype. Regarding the intrauterine diagnosis of hemoglobin SS (HbSS), 20.0% would allow pregnancy, 31.7% would abort, and 48.3% were undecided. Most respondents (73.2%) had good knowledge of SCA (mean score = 7.0/10.0). If partners were found to have SCA after marriage, 42.9% were undecided. Knowledge of SCA was significantly associated with sex (p = 0.017) and education level (p = 0.001).

Conclusion

Blood donors in Ibadan have good knowledge, awareness, and attitudes toward SCA. However, more health education would benefit the population.

## Introduction

Sickle cell anemia (SCA) is a complex genetic condition caused by the presence of homozygous mutant hemoglobin SS genes (HbSS) [1]. About 300,000 babies with SCA are born globally each year, most of them in Africa. More than half of these African children will die before the age of 5. Nigeria has the highest burden of the disease globally, affecting about 2% of its population [1,2]. Recent improvements in diagnosis and advancements in the management of SCA in children have led to better survival rates and an increased number of adult patients who require optimal care for the complications and morbid states associated with the condition [3-5].

The mutation in the hemoglobin gene causes crystallization and polymerization of hemoglobin, disrupting RBC structure and flexibility. These effects are responsible for the various crises in SCA and reduce the lifespan of the RBC to about 25 days in SCA patients [6,7]. One intervention to prevent the complications of SCA is by reducing the rate of HbS polymerization. Different therapeutic approaches following this mechanism have been developed, such as gene therapy, hematopoietic stem cell transplantation, the use of hydroxyurea, antioxidant, and anti-adhesive therapy. Several other interventions have been implemented, but their unwanted side effects, associated toxicities, questionable efficacy, and success rates have limited their optimal use [8-11]. However, blood transfusion is another therapeutic option with manageable complications.

Research has shown that blood transfusion is one of the mainstays of treatment for most of the severe acute crises seen in SCA. It increases the oxygen-carrying capacity of blood and reduces the complications of vaso-occlusion [12,13]. However, this is affected by ineffective blood banking services and the apathy of blood donors towards donating blood. In a study by [7], 75% of hospitals in Nigeria were unable to transfuse patients regularly due to blood scarcity, which is largely due to low availability and limited donors. It was also reported that a shortage of blood supply among other factors has limited the care of SCA patients [3,7].

With the high prevalence of SCA in Nigeria, there is a need to focus on primary prevention through public health education and enlightenment. This is crucial for individuals to make informed decisions about their reproductive life and other health-related choices [14]. This study, hence, seeks to assess the level of knowledge and attitudes of blood donors in Ibadan, Nigeria, towards SCA patients. It also aims to evaluate the relationship between the socio-demographic characteristics (sex, occupation, and level of education) of the blood donors and their knowledge about SCA.

This article was previously presented as an abstract poster at the International Research Centre of Excellence 2nd Annual Scientific Symposium on August 14, 2024.

## Materials and methods

Study design

The study was a descriptive cross-sectional study conducted among blood donors at the University College Hospital Blood Bank, Ibadan. It was carried out over three months, from April to June 2023.

Study area

The University College Hospital Blood Bank is one of the largest and most recognized blood banks in Ibadan, Nigeria. Located on the premises of the University College Hospital, the premier federal tertiary teaching hospital in Ibadan, the blood bank serves not only the hospital's patients but also those from several other hospitals in the city. Annually, the blood bank collaborates with various organizations, including the SmileBuilders Initiative, to organize several blood donation drives, such as the Donate-A-Pint project, where blood donors voluntarily donate blood.

Study population

For this study, the subjects included blood donors at the Donate-A-Pint project around the University College Hospital, Ibadan. After providing consent, participants were asked to fill out a printed questionnaire.

Inclusion criteria

Consenting blood donors for the Donate-A-Pint project at the donation site were included.

Exclusion criteria

Any donor outside the Donate-A-Pint project and those who refused to consent were excluded.

Sample size determination

The sample size for this study was estimated using the Yamane formula. The average monthly donor count at the University College Hospital Blood Bank over the last six months is 810. Using the Yamane formula: n = N / (1 + Ne^2); where n = sample size for your time frame, N = population size (estimated monthly donor count), and e = tolerance error. With a 10% contingency, the final minimum sample size (n) for this study was estimated to be 295 blood donors.

Sampling technique

All the blood donors who consented to participate were provided with a printed questionnaire to fill out at the blood donation locations.

Data collection

Data were collected using a self-administered validated printed questionnaire written in English, the official and widely spoken language at the study locations. It was also translated into Yoruba, the native language in Ibadan, Nigeria. Volunteers in the Donate-A-Pint Project made efforts to explain the questionnaire to participants who were unclear about parts of it and to ensure the forms were appropriately filled and collected before participants left the blood bank, to ensure optimal compliance. Two hundred and five (205) questionnaires were retrieved and subjected to statistical analysis.

Study tool

The structured self-administered questionnaire was designed and validated using questions from similar studies conducted in Nigeria [15,16]. The questionnaire comprised 23 questions divided into four sections: socio-demographic characteristics, awareness, attitude, and knowledge of the respondents. The socio-demographic section included the respondent’s age, gender, level of education, and religion. The knowledge section included questions on the common symptoms, diagnosis, treatment, and prevention of SCA, which were answered as either ‘Yes,’ ‘No,’ or ‘I don’t know.’ The respondents' attitudes were assessed using a three-point scale with responses such as ‘Yes,’ ‘No,’ and ‘Undecided.’ The questions addressed the objectives of the study. The questionnaire was pretested among 20 randomly selected blood donors from the previous donation drive to test the feasibility and efficiency of the questionnaire, and the data collected were not included in the study.

Data analysis

The data for the study were entered into a password-protected computer system, and the respondents' information was handled with confidentiality. Data were entered into Microsoft Office Excel 2016 and analyzed using the SPSS version 23. Descriptive analysis for categorical data was presented as frequencies and percentages, and continuous data were presented in means, standard deviations, and ranges. The Chi-squared test was used to assess the relationship between sociodemographic factors (sex, age range, education) and the level of knowledge, as well as the relationship between awareness and attitudes towards SCA. A p-value of ≤ 0.05 at a 95% CI was considered statistically significant.

## Results

Sociodemographic features of study participants

A total of 205 respondents participated in the study. Deferred donors were not included. The majority of the respondents were male (141, 68.8%) and were within the age category of ≥25 years (87, 42.4%). Of the studied population, 78.5% (161) were single, and 60% (123) were students. Most (140, 68%) of the respondents were voluntary blood donors, and 71.7% (147) were currently attaining tertiary education. Table 1 summarizes the socio-demographic features of the respondents.

**Table 1:** Sociodemographic features of respondents.

Characteristics of blood donors	Frequency	Percentage (%)
Age (years)		
<18	4	2
19-21	29	14.1
22-24	85	41.5
≥25	87	42.4
Gender		
Male	141	68.8
Female	64	31.2
Level of Education		
Primary	1	0.5
Secondary	30	14.6
Tertiary	147	71.7
Post-graduate	25	12.2
Marital Status		
Single	161	78.5
Engaged	5	2.4
Married	36	17.6
Divorced	3	1.5
Occupation		
Student	123	60
Non-student/Professional	82	40
Religion		
Christianity	148	68.3
Traditional	2	1
Reasons for donating		
Voluntary	140	68.3
Involuntary	54	23.3
Others	11	5.4

Awareness of blood donors to SCA

Most (79.5%) of the respondents knew their genotype, and the majority (55.1%) had the Hb AA genotype. 87.8% had heard about sickle cell disease, and 52.2% knew people living with the condition. Moreover, 78.5% had correct knowledge of SCA being an inherited disorder. The summary of questions asked to demonstrate respondents' awareness of sickle cell disease is depicted in Table 2.

**Table 2:** Awareness of sickle cell anemia. N: Number of respondents; SCA: Sickle cell anemia.

Variables	N	Percentage (%)
Do you know your genotype?		
No	42	20.5
Yes	163	79.5
What is your genotype?		
AA	113	55.1
AS	41	20
AC	12	5.9
Not Applicable	39	19
Have you heard of SCA?		
No	25	12.3
Yes	180	87.8
Do you know anyone living with SCA?		
No	98	47.8
Yes	107	52.2
Is SCA an inherited disorder		
No	44	21.5
Yes	161	78.5

Attitude of blood donors to SCA

The majority (86.3%) of the respondents were willing to opt for genetic counseling as a necessary precautionary attitude towards SCA. Most (73.2%) of the respondents were not willing to continue a relationship after discovering that they were at risk of having offspring with SCA, while 7.8% were willing to continue the relationship, and 19% were undecided. Almost 43% of the respondents were undecided on the action to take if they discovered that they or their partner has SCA after marriage.

Also, 48.3% of the respondents were unsure of what they would do if they discovered that their unborn child has SCA following diagnosis, and 62.4% felt empathetic when they saw people living with SCA. The attitude of respondents towards sickle cell disease is as shown in Table 3.

**Table 3:** Attitude towards sickle cell anemia. N: Number of respondents; SCA: Sickle cell anemia.

Variables	N	Percentage (%)
Are you willing to do genetic counseling?		
Yes	177	86.3
No	11	5.4
Not Applicable	17	8.3
Are you willing to continue with a relationship despite the risk of having offspring with SCA?		
Yes	16	7.8
No	150	73.2
Undecided	39	19
What will you do if you and your partner are found to be SCA after marriage?		
Opt for prenatal screening	70	34.1
Accept not to have biological children	37	18
Accept to have children with SCA	10	4.9
Undecided	88	42.9
What will you do if your unborn child has SCA following diagnosis?		
Abortion	65	31.7
Allow pregnancy	41	20
Undecided	99	48.3
How do you feel when you see someone with SCA?		
Worried	53	25.9
Empathetic	128	62.4
Indifferent	24	11.7

Knowledge of blood donors about SCA

More than 80% of the respondents know that bone pain is a common symptom of SCA. 71.7% know that yellowness of the eyes is a common symptom, 71.2% know that delayed growth is a common symptom, 59.5% know that swelling of hands and feet is a common symptom, and 82% know that easy fatiguability is a common symptom of SCA. About 83% believed that blood samples could be used to screen for SCA, while half (50.2%) did not know whether urine samples could be used for screening. Most (83.9%) respondents believed that anemia can be managed with medication and lifestyle changes, and 86.3% believed that premarital genotype screening can help in reducing the prevalence of SCA. Table 4 summarizes the level of knowledge of respondents about SCA.

**Table 4:** Knowledge of sickle cell anemia. N: Number of respondents.

Variables	N	Percentage
Bone pain is a common symptom of sickle cell anemia		
Yes	166	81
No	11	5.4
I don’t know	28	13.7
Yellowness of the eyes is a common symptom of sickle cell anemia		
Yes	147	71.7
No	14	6.8
I don’t know	44	21.5
Delayed growth is a common symptom of sickle cell anemia		
Yes	146	71.2
No	23	11.2
I don’t know	36	17.6
Swelling of hands and feet is a common symptom of sickle cell anemia		
Yes	122	59.5
No	18	8.8
I don’t know	65	31.7
Easy fatiguability is a common symptom of sickle cell anemia		
Yes	168	82
No	6	2.9
I don’t know	31	15.1
Blood samples can be used to screen for sickle cell anemia		
Yes	170	82.9
No	7	3.4
I don’t know	28	32.7
Urine samples can be used to screen for sickle cell anemia		
Yes	51	24.9
No	51	24.9
I don’t know	103	50.2
There is no known effective cure for sickle cell anemia		
Yes	114	55.6
No	44	21.5
I don’t know	47	22.9
Sickle cell anemia can be managed with medication and lifestyle change		
Yes	172	83.9
No	10	4.9
I don’t know	23	11.2
Premarital genotype screening can help in reducing the prevalence of sickle cell anemia		
Yes	177	86.3
No	7	3.4
I don’t know	21	10.2

## Discussion

This study assessed the level of awareness, knowledge, and attitude of blood donors in Ibadan and their associations. Our analysis suggests that most of the donors had good knowledge of SCA, were aware of SCA, and showed a positive attitude towards SCA.

Our finding shows that 73.2% of the participants had a good knowledge of SCA. This finding is consistent with studies conducted among similar participants across universities in the country, where a good knowledge of SCA was reported [17,18]. This result is expected, given that the participants are educated and have access to ample resources to acquire such knowledge. However, several other studies reported a low percentage of participants with good knowledge of SCA [14,19-22]. While the difference in the reported level of knowledge could be due to differences among the groups and the assessment criteria, these people could be SCA carriers and are at risk of giving birth to children with SCA [20].

We found that most of our participants were aware of SCA, and most (86.3%) were willing to perform genetic counseling. This agrees with the findings of similar studies where 77% and 85.4% of respondents, respectively, agreed to consider genetic counseling before marriage [16,20]. However, a significant percentage of the participants (42.9%) were undecided on what to do if either of their partners were found to have SCA after marriage. This is in contrast with other studies where most participants were willing to call off their marriages if discovered that their genotypes predispose them to having children with SCA [16,23].

Also, most of the participants (48.3%) were undecided on what to do if their unborn child has SCA following diagnosis. A similar occurrence was reported in a study where all the participants (100%) were undecided on what to do in such a situation [16]. This might be because participants were unaware of the benefits of opting for prenatal screening, the legislation on the abortion of babies with SCA, and the otherwise painful and psychosocial trauma that affected children experience. Additionally, the 5.4% of participants who were unwilling to undergo genetic counseling and the 8.3% who were undecided emphasize the need for public enlightenment in the primary prevention of SCA.

Findings from this study revealed that the participants’ age, religion, and occupation did not have a significant association with their level of knowledge about SCA. On the other hand, participants’ sex and level of education have significant associations. This is consistent with similar studies in Ghana where the level of knowledge of SCA was significantly associated with the level of education [20,23] and the sex of participants [23]. However, a study conducted on undergraduates in Lagos reported no association with the level of knowledge of participants. Our study revealed a better knowledge score amongst male participants (46.3%), as compared to females (26.6%). This could be due to the larger number of male participants in the study. A similar finding was reported in a cross-sectional study amongst unmarried adults in Nigeria’s capital city [24].

A limitation of this study is the small sample size, which may make generalizability questionable. The use of closed questions requiring respondents to choose among the options did not permit them to explain their answers, hence introducing some bias. The respondents of the study were mainly students and residents around the blood bank; hence the results may not accurately reflect the awareness, knowledge, and attitude towards SCA among the broader population of Ibadan residents.

## Conclusions

Most participants were aware of SCA. To ensure appropriate decisions and steps are taken on SCA-related issues, more efforts should be directed at public health education on SCA management. There is a need for sustained mass motivational campaigns on the importance of genetic counseling and blood donation among students, youths, and the general public. We recommend conducting regular continuous medical education and seminars on SCA to bridge knowledge gaps, dispel misconceptions, and promote efforts to control SCA.
